# Massive Lower Gastrointestinal Bleeding From Colonic Submucosal Lipoma and Diverticular Disease: A Rare Case Requiring Surgical Interventions and Postoperative Management Challenges

**DOI:** 10.7759/cureus.82133

**Published:** 2025-04-12

**Authors:** Sandeepa D Dadigamuwage, Alexander Macaulay, Mafaiz Jaufer

**Affiliations:** 1 Colorectal Surgery, University Hospitals Plymouth NHS Trust, Plymouth, GBR; 2 Radiology, University Hospitals Plymouth NHS Trust, Plymouth, GBR

**Keywords:** colonic diverticular disease, colonic submucosal lipoma, emergency hemicolectomy, lower gastrointestinal bleeding, massive transfusion protocol

## Abstract

Massive lower gastrointestinal (GI) bleeding (LGIB) is a life-threatening condition requiring prompt diagnosis and management. While diverticular disease is a common cause, rare entities such as colonic submucosal lipomas may complicate the presentation. This case highlights the diagnostic and therapeutic challenges in managing such a scenario, particularly with recurrent bleeding and postoperative complications. A 61-year-old patient presented with recurrent episodes of massive LGIB over a five-day period. Initial evaluations, including CT angiography and nuclear medicine imaging, identified diverticular disease but failed to localize the bleeding source definitively. Despite receiving 15 units of blood transfusions, the patient experienced persistent bleeding, ultimately necessitating emergency surgery. A right hemicolectomy revealed a submucosal lipoma and diverticular disease in the ascending colon with significant intraluminal hemorrhage. Postoperative management was complicated by pulmonary embolism, requiring therapeutic anticoagulation. This case emphasizes the importance of integrating multiple diagnostic modalities when evaluating massive LGIB, especially in patients with uncommon etiologies. It also highlights the complexities of managing massive transfusion protocols and postoperative thromboembolic events in high-risk patients. This report underscores the need for timely surgical intervention in unresolved cases of massive LGIB and the importance of vigilant postoperative care to prevent complications. Enhanced strategies for early mobilization and hydration are critical for improving outcomes in these patients.

## Introduction

Lower gastrointestinal (GI) bleeding can cause significant mortality and morbidity, leading to prolonged hospital stays, multiple blood transfusions, and surgical or radiological interventions [1]. Lower GI bleeding (LGIB) is defined as bleeding from a lesion distal to the ligament of Treitz, including the small and large bowel [2]. All patients should undergo routine observations, a complete history, and an examination, including a digital rectal examination and appropriate haematological investigations. The shock index, calculated by dividing the heart rate by the systolic blood pressure, is a useful marker of hemodynamic instability in the setting of active haemorrhage [3].

LGIB arises from diverse causes, including degenerative conditions such as diverticular disease, malignancies (e.g., colorectal carcinoma, GI stromal tumours), anticoagulation therapy, angiodysplasia, infectious diseases, and inflammatory bowel diseases, such as Crohn's disease or ulcerative colitis. Symptoms range from heavy per rectal bleeding to minimal occult bleeding, with bright red bleeding typically indicating anorectal sources and altered blood suggesting bleeding above the rectosigmoid junction. Right colonic bleeding may present with anaemia-related symptoms, including exertional fatigue and dizziness. Initial assessment involves detailed history-taking, including family history of colorectal cancer and inflammatory bowel disease risk, and documenting anticoagulation use to guide therapeutic modifications. Thorough clinical examination, including digital rectal examination, is essential to identify the source and severity of bleeding. Colonic lipomas are rare benign tumors, with symptomatic presentations such as massive LGIB being particularly uncommon. For instance, Bahadursingh et al. reported a case of a giant submucosal sigmoid colon lipoma presenting with significant bleeding [4].

Abdominal and digital rectal examinations should be performed for all patients presenting with LGIB. If available, proctoscopy should also be considered. Abdominal examination may reveal tenderness, distension, or a mass, depending on the underlying cause. During the digital rectal examination, assess for haematochezia and anorectal pathology, such as haemorrhoids. Studies have shown that left colonic bleeding tends to be bright red, whereas right colonic bleeding is usually maroon and may be associated with clots, which typically indicate a bleeding source above the anal canal, where blood can accumulate and clot over time [5].

Initial management of per rectal bleeding prioritizes resuscitation with crystalloids and blood product transfusions as needed. Essential haematological investigations include full blood count, renal and liver function tests, coagulation profiles, and blood grouping, with advanced tests such as rotational thromboelastometry (ROTEM) for suspected coagulation disorders. Colonoscopy, capable of identifying the bleeding source in over 75% of cases and offering therapeutic options, is recommended within 24 hours of admission. However, it may be limited during active bleeding and in an unprepared bowel due to poor visualization. In such cases, local interventions such as adrenaline injection or radiological modalities can provide alternative diagnostic and therapeutic approaches [5].

Computed tomographic angiography (CTA) can detect active GI bleeding at rates as low as 0.3 mL/min, with a reported sensitivity of approximately 85% [6]. Catheter angiography, which has lower sensitivity than CT angiography, is typically reserved for hemodynamically unstable patients when concurrent embolisation is planned. It serves as both a diagnostic and therapeutic tool in managing ongoing or recurrent bleeding. Radionuclide imaging, using Technetium-99m-based tracers, is helpful for detecting intermittent, scant bleeding. The long half-life of Technetium-99m allows for repeated scans within a 24-hour period to obtain sequential images. Surgery may be required if radiological and endoscopic procedures fail. It is advisable to consult a colorectal surgeon early in the diagnostic process, as these patients can deteriorate rapidly. In cases of hemodynamic instability requiring more than six units of blood within 24 hours and failing to respond to resuscitation, emergency segmental resection or subtotal colectomy may be necessary [5].

## Case presentation

A 61-year-old male patient, with a known history of hypertension, presented to the acute surgical assessment unit following one episode of painless per rectal bleeding. He had a history of haemorrhoids 20 years ago, which were managed conservatively. The patient described the current bleeding as altered red in color with the passage of clots, estimating the volume to be approximately 300-400 mL. The bleeding was not associated with abdominal pain, fever, or changes in bowel habits. The patient's family history was negative for bowel cancers or inflammatory bowel diseases, and he was not taking any anticoagulation medications. On examination, both the abdominal and digital rectal examinations (DRE) were unremarkable.

Initial haematological investigations revealed the following findings (Table 1).

**Table 1:** Haematological investigation results on admission CRP: C-Reactive Protein; eGFR: Estimated Glomerular Filtration Rate; APTT: Activated Partial Thromboplastin Time; PT: Prothrombin Time; INR: International Normalization Ratio; MCV: Mean Cell Volume

Parameter	Results	Reference Range
eGFR	>90	≥ 90 mL/min/1.73m^2^
CRP	3	0.1-5 mg/L
Sodium	135	133-146 mmol/L
Potassium	3.5	3.5-5.3 mmol/L
Urea	5.5	2.5-7.8 mmol/L
Creatinine	60	64-104 mmol/L
APTT ratio	0.9	N/A
APTT	26.3	24-34 seconds
PT	13.5	12.5-15.5 seconds
INR	1.0	N/A
White Cells	10.2	3.6-9.2x10^9^/L
Haemoglobin	72	130-175 g/L
Haematocrit	20.1	%
MCV	88.2	80-105 fL
Platelets	340	150-450x10^9^/L
Neutrophils	7.9	1.7-6.2x10^9^/L

Venous blood gas analysis demonstrated the following results (Table 2).

**Table 2:** Blood gas analysis results on admission pH: Potential of Hydrogen; PaCO_2_: Partial Pressure of Carbon Dioxide; PaO_2_: Partial Pressure of Oxygen; SaO_2_: Oxygen Saturation in Blood

Parameter	Results	Reference range
pH	7.42	7.35-7.45
PaCO_2_	4.87	4.7-6.0 kPa
PaO_2_	4.76	10-13 kPa
Haemoglobin	119	130-175 g/L
SaO_2_	68.8	%
Ionized Calcium	1.14	1.12-1.32 mmol/L
Lactate	1.9	0.5-2.0 mmol/L
Bicarbonate	23.5	22-26 mmol/L

The patient received one unit of blood transfusion during the initial admission, which raised his hemoglobin to 84 g/L. As there were no further episodes of bleeding, he was discharged with a follow-up plan for outpatient colonoscopy under the two-week-wait (2WW) pathway. Although a formal risk scoring system was not applied at the time, based on clinical stability, absence of ongoing bleeding, and reassuring lab parameters, the patient would retrospectively meet low-risk criteria per the Oakland score, which is endorsed by the British Society of Gastroenterology.

However, the patient was readmitted to the surgical assessment unit three days after discharge with a recurrence of per rectal bleeding. He reported dizziness but denied any shortness of breath. On examination, the patient appeared pale, with an unremarkable abdominal examination. DRE revealed altered blood on the gloved finger. The haematological investigations following re-admission demonstrated the following findings (Table 3).

**Table 3:** Haematological investigation results on re-admission CRP: C-Reactive Protein; eGFR: Estimated Glomerular Filtration Rate; APTT: Activated Partial Thromboplastin Time; PT: Prothrombin Time; INR: International Normalization Ratio; MCV: Mean Cell Volume

Parameter	Results on admission	Reference range
eGFR	>90	≥ 90 mL/min/1.73m^2^
CRP	1	0.1-5 mg/L
Sodium	142	133-146 mmol/L
Potassium	3.3	3.5-5.3 mmol/L
Urea	6.9	2.5-7.8 mmol/L
Creatinine	80	64-104 mmol/L
APTT ratio	0.8	N/A
APTT	24.7	24-34 seconds
PT	13	12.5-15.5 seconds
INR	0.9	N/A
White Cells	14.8	3.6-9.2x10^9^/L
Haemoglobin	70	130-175 g/L
Haematocrit	19.9	%
MCV	91.4	80-105 fL
Platelets	329	150-450x10^9^/L
Neutrophils	9.7	1.7-6.2x10^9^/L

A stool sample was sent for bacteriology, and the findings showed the following results (Table 4).

**Table 4:** Stool sample results Spp: Species; PCR: Polymerase Chain Reaction; E.coli: Escherechia coli; EIEC: Enteroinvasive Escherechia coli; C.diff: Clostridioides difficile; GDH: Glutamate Dehydrogenase; ELISA: Enzyme-Linked Immunosorbent Assay

Test	Results
Campylobacter spp PCR	Negative
Salmonella spp PCR	Negative
Shiga toxin E. coli PCR	Negative
Shigella spp EIEC PCR	Negative
C. Diff GDH	Negative
Cryptosporidium/Giardia ELISA	Negative

The patient received two units of blood transfusion, which raised his haemoglobin to 84 g/L. However, later that day, he experienced a Medical Emergency Team (MET) call after becoming dizzy and suffering a fall. He was hypotensive with a transient loss of consciousness lasting three minutes. Repeat haematological investigations at the end of the day revealed a haemoglobin level of 70 g/L, a pH level of 7.18, and a lactate level of 14.4. He was administered two more units of blood transfusion along with fluid resuscitation, but his haemoglobin remained low at 77 g/L, raising concerns of ongoing bleeding. The patient was started on 1 g of tranexamic acid three times a day. At this point, the patient’s shock index was 1.06, further supporting the presence of hemodynamically significant ongoing bleeding.

**Figure 1:**
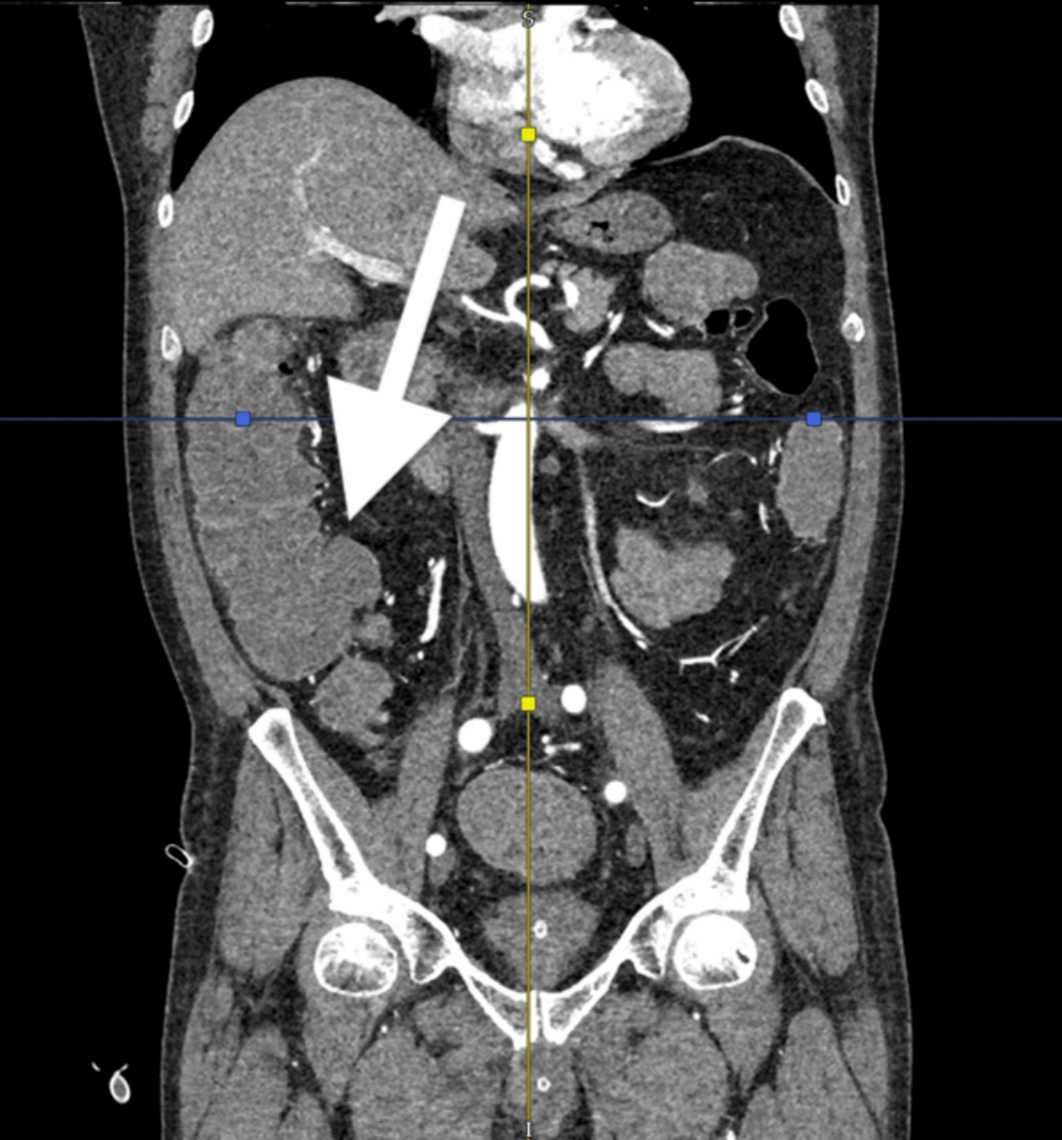
Portal venous phase coronal section of a CT angiogram demonstrating diverticulosis of the ascending colon but no contrast extravasation

A CT angiogram was performed, which did not demonstrate any active bleeding or lesions in the bowel. Following the transfusion of another two units of blood, his haemoglobin improved to 92 g/L. However, the next morning, the patient experienced another MET call due to hypotension and a large episode of per rectal bleeding, with a haemoglobin level dropping to 75 g/L. He was given two more units of blood transfusion. The case was discussed with the haematology team regarding blood product transfusion. As clotting studies were within normal limits, it was decided not to administer additional blood products apart from blood transfusion. The following day, his haemoglobin dropped to 64 g/L, prompting the transfusion of three more units of blood. An upper gastrointestinal (GI) endoscopy (OGD) was performed, which revealed a few fundic gland polyps but was otherwise grossly normal. A repeat CT angiogram showed mild, uncomplicated diverticulosis throughout the colon but no evidence of active bleeding or bowel wall thickening (Figure 1).

**Figure 2:**
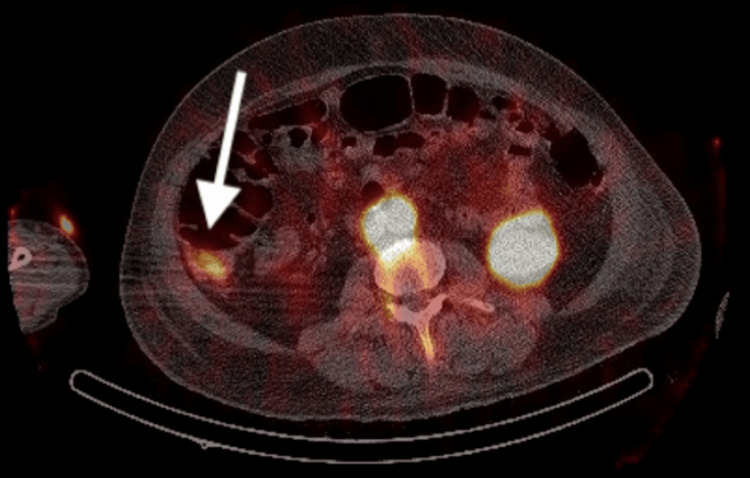
Axial section of a Technetium-99m labelled red blood cell SPECT-CT study demonstrating radionucleide accumulation within the ascending colon

Later that day, the patient experienced another MET call with hypotension and a significant drop in haemoglobin to 75 g/L. He received four units of blood transfusion and two units of cryoprecipitate as per haematological advice. Despite normal clotting studies, he was referred to the Intensive Treatment Unit (ITU), and a Rotational Thromboelastometry (ROTEM) study was conducted. The ROTEM results were normal, but his ionized calcium levels were slightly low and subsequently corrected. A red cell-labelled nuclear medicine scan was performed, but no bleeding was observed during the 40-minute post-injection period. The following day, his haemoglobin dropped to 77 g/L, prompting the administration of two more units of blood transfusion and two units of cryoprecipitate. Later that day, a repeat red cell-labelled nuclear medicine scan demonstrated a faint blush of activity in the right mid-abdomen laterally, raising suspicion of a bleeding point in the ascending colon (Figure 2).

**Figure 3:**
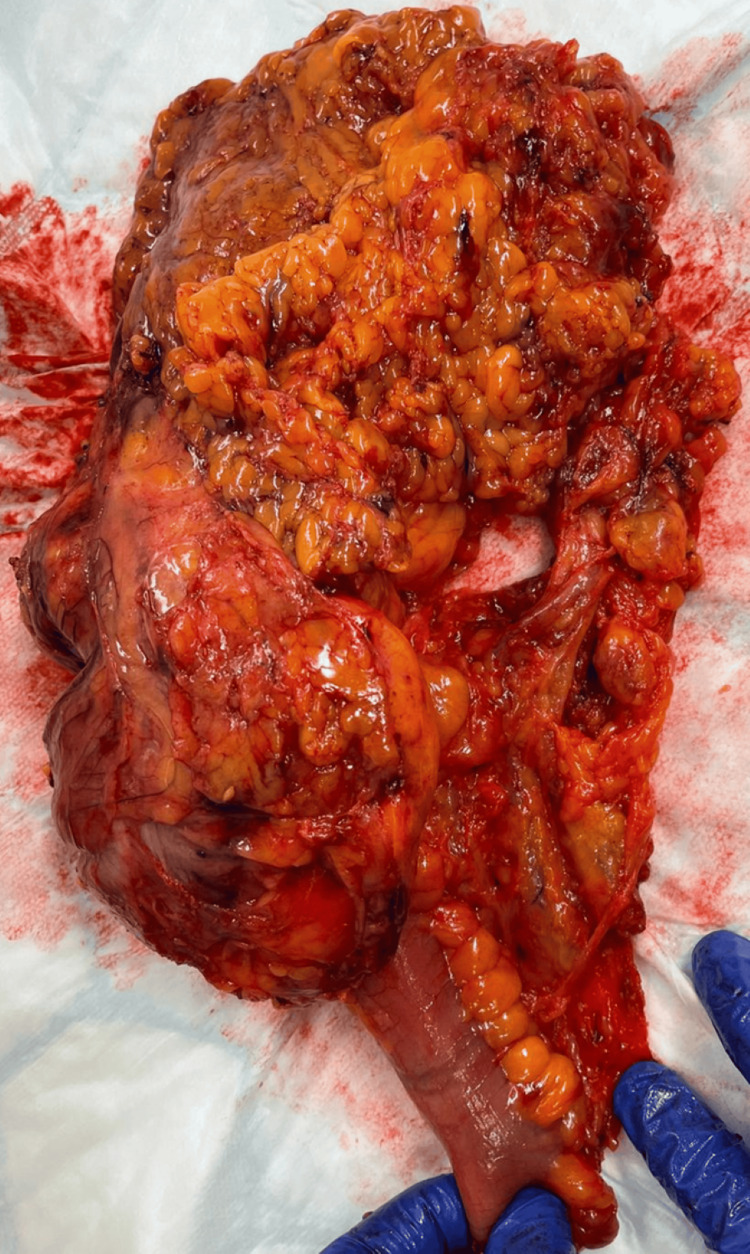
Resected specimen from right hemicolectomy

Despite these findings, the patient experienced another significant bleeding episode the next day, with his haemoglobin dropping to 55 g/L. He was transfused with four units of blood, and the decision was made to proceed with surgery due to the severity and persistence of his bleeding. The patient underwent an emergency laparotomy, right hemicolectomy with the formation of a double-barrel stoma to monitor any ongoing bleeding. The resected specimen (Figure 3) was sent for histological analysis.

**Figure 4:**
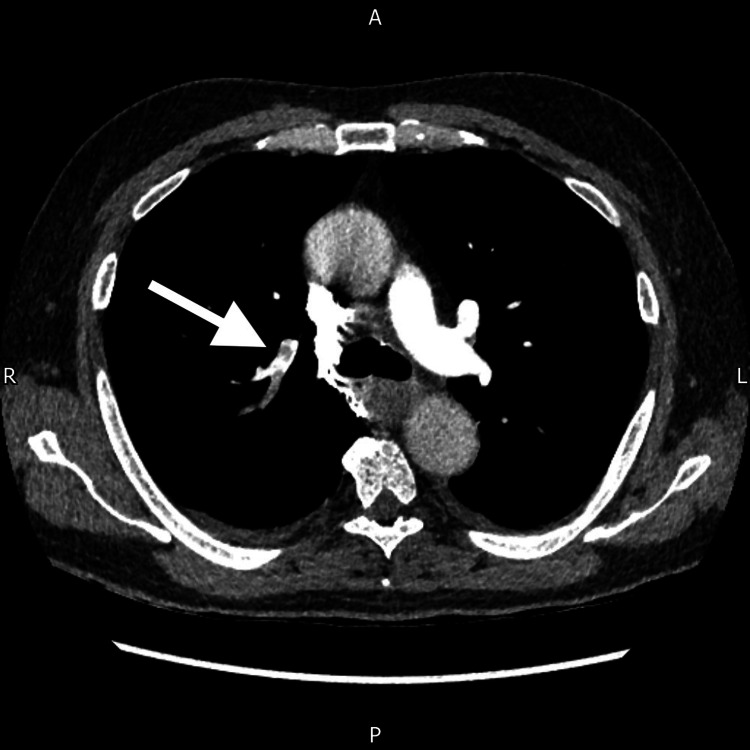
CT pulmonary angiogram, shown in an axial cut, demonstrating a pulmonary embolus in the right lung

Postoperatively, his haemoglobin levels stabilized within the range of 80-100 g/L, and he did not require further transfusions of blood or blood products. Histological examination of the specimen confirmed intraluminal haemorrhage arising from both a submucosal lipoma in the ascending colon and underlying colonic diverticular disease. There was no evidence of dysplasia or malignancy. On the third postoperative day, the patient developed hypoxia with a new oxygen requirement. A CT pulmonary angiogram confirmed the presence of multiple pulmonary emboli with acute segmental and subsegmental involvement in the right upper and lower lobes (Figure 4). He was initiated on therapeutic anticoagulation with low molecular weight heparin (LMWH). Pharmacological thromboprophylaxis was initially withheld due to the high risk of ongoing gastrointestinal bleeding, despite the patient’s elevated risk for venous thromboembolism.

The patient recovered without further complications and was discharged on the 19th day following his second admission. The transfusion and treatment summary is presented in Table 5.

**Table 5:** Patient transfusion and treatment summary

Admission Phase	Hemoglobin Level (g/L)	Transfusions Given	Intervention
First Admission	72 (on admission)	None	Discharged with outpatient colonoscopy plan
Second Admission (Recurrent Bleeding)	116 → 84	2 units of blood → later 2 more units (Total: 4)	Admitted, MET call due to dizziness and hypotension
Continued Bleeding and Escalation	75 → 64	2 more units → 3 more units (Total: 9 cumulative)	Hemodynamic instability, recurrent bleeding
Intensive Care and Further Decline	77 → 55	2 more units + 2 units of cryoprecipitate → later 4 more units (Total: 15 cumulative)	Referred to ITU, ROTEM study normal
Surgical Intervention	80–100 (post-surgery)	No further transfusions required	Emergency laparotomy and right hemicolectomy

## Discussion

LGIB is relatively common, accounting for 20%-30% of all cases of major GI bleeding. The incidence is higher in older patients and those on multiple medications or polypharmacy. Approximately 80%-85% of LGIB originate distal to the ileocecal valve, while only 0.7%-9% originate from the small intestine [5]. This is consistent with data from Strate et al. [1], who report similar prevalence rates in large-scale studies. The increased risk in older populations and those with polypharmacy is widely supported in the literature, reflecting age-related vascular fragility and medication-induced mucosal injury.

LGIB can be categorized as massive, moderate, or occult. Massive bleeding typically presents with hypotensive shock and large volumes of rectal bleeding, often necessitating multiple blood transfusions and urgent intervention. The most common causes include diverticular disease and angiodysplasia. This aligns with findings from Oakland et al., who highlight these conditions as leading causes of significant bleeding [3]. Moderate bleeding is associated with conditions such as neoplastic diseases, inflammatory bowel disease, infectious causes, iatrogenic causes such as post-polypectomy, benign anorectal conditions, and congenital disorders. These patients are often hemodynamically stable, a characteristic well-supported by studies on endoscopic findings and interventions [7]. Occult bleeding, on the other hand, can occur at any age and typically presents with symptoms of chronic anaemia, consistent with studies that describe it as a leading cause of iron-deficiency anaemia [5].

Various endoscopic and radiographic modalities are available for the evaluation and treatment of LGIB, including flexible sigmoidoscopy, colonoscopy, angiography, radionuclide scintigraphy, and multi-detector row computed tomography (CT) [8]. While no single modality is universally considered the gold standard, colonoscopy remains the preferred initial diagnostic and therapeutic tool for most patients presenting with haematochezia suspected to originate from the colon [9]. However, in cases of active bleeding, colonoscopy can be challenging due to the unprepared bowel, which increases the risk of iatrogenic injury caused by poor visualization. These challenges have been highlighted by Green et al. [7], who noted similar limitations in their analyses [7]. Additionally, bowel preparation can exacerbate electrolyte imbalances and fluid depletion in patients already at risk of hypovolemic shock. Sedation during the procedure can also negatively impact the patient’s physiology, delaying recovery. In this case, colonoscopy was not performed due to these concerns. Instead, the positive findings on the nuclear medicine scan guided the decision to proceed with surgery.

Bleeding from colonic lipomas is an uncommon but recognized complication. Proposed mechanisms include mucosal ulceration overlying the lipoma, pressure necrosis, or erosion into submucosal vessels, all of which can result in significant haemorrhage. In our case, the lipoma was submucosal and located in the ascending colon, contributing to intraluminal bleeding. Preoperative imaging, including CT angiography and radionuclide scanning, failed to detect the lesion, likely due to the absence of contrast extravasation and the soft-tissue density of lipomas, which can be easily overlooked or mistaken for surrounding bowel content, especially during active bleeding. This highlights the diagnostic challenge and reinforces the need for surgical exploration when bleeding is severe and recurrent, despite non-diagnostic imaging.

Massive transfusion (MT) is a lifesaving intervention for haemorrhagic shock but carries significant risks. The lethal triad of acidosis, hypothermia, and coagulopathy associated with MT contributes to high mortality rates. Other complications include acid-base disturbances, hypothermia, electrolyte abnormalities (e.g., hypocalcaemia, hypomagnesemia, hypokalaemia, hyperkalaemia), citrate toxicity, and transfusion-associated acute lung injury [10]. The findings in this case align with those of Spinella et al., who emphasize the critical risks associated with MT in patients undergoing MT protocols [11].

Despite receiving prophylactic doses of low molecular weight heparin (LMWH) and thrombo-embolic deterrent (TED) stockings, the patient developed postoperative pulmonary embolism. This highlights the importance of early mobilization and maintaining adequate hydration to prevent thromboembolic events, particularly in patients who have undergone MT or recent major abdominal surgery. This finding is consistent with evidence from Monreal et al., who underscore delayed mobilization and systemic inflammation as key contributors to thromboembolic risk [12].

## Conclusions

This case highlights a rare but important cause of massive LGIB due to a colonic submucosal lipoma in the presence of diverticular disease. The diagnostic challenges presented by negative imaging and inconclusive endoscopic findings underscore the importance of maintaining a broad differential and considering surgical intervention when conservative measures fail. In this patient, emergency laparotomy and right hemicolectomy provided definitive management, with histology confirming intraluminal hemorrhage from both the lipoma and diverticular disease.

The postoperative course was complicated by pulmonary embolism despite standard thromboprophylaxis, emphasizing the heightened risk in patients undergoing major abdominal surgery with massive transfusion. This case reinforces the need for early mobilization, vigilant monitoring, and individualized care plans. Clinicians should also consider rare bleeding sources in recurrent or unexplained lower GI hemorrhage, and multidisciplinary collaboration remains critical for timely decision-making and optimal outcomes.
